# Parental Attitudes and Perceptions of Support after Brief Clinician Intervention Predict Intentions to Accept the Adjuvanted Seasonal Influenza Vaccination: Findings from the Pediatric Influenza Vaccination Optimization Trial (PIVOT)–I

**DOI:** 10.3390/vaccines10111957

**Published:** 2022-11-18

**Authors:** William A. Fisher, Vladimir Gilca, Michelle Murti, Alison Orth, Hartley Garfield, Paul Roumeliotis, Emmanouil Rampakakis, Vivien Brown, John Yaremko, Paul Van Buynder, Constantina Boikos, James A. Mansi

**Affiliations:** 1 Department of Psychology, Department of Obstetrics and Gynaecology, Western University, London, ON N6A 3K7, Canada; 2 Département de Médecine Sociale et Préventive, Faculté de Médecine, Institut Nationale de Sante Publique du Québec and Université Laval, Québec City, QC G1V 5B3, Canada; 3 Dalla Lana School of Public Health, University of Toronto, Toronto, ON M5T 3M7, Canada; 4 Fraser Health Authority, Vancouver, BC V3T 0H1, Canada; 5 The Hospital for Sick Children, University of Toronto, Toronto, ON M5G 1X8, Canada; 6 Eastern Ontario Health Unit, Cornwall, ON K6J 5T1, Canada; 7 JSS Medical Research, Montreal, QC H4S 1N8, Canada; 8 Department of Family and Community Medicine, University of Toronto, Toronto, ON M5G 1V7, Canada; 9 The Montreal Children’s Hospital, Montreal, QC H4A 3J1, Canada; 10 Department of Pediatrics, McGill University, Montreal, QC H3A 0G4, Canada; 11 School of Medicine, Griffith University, University of Western Australia, Perth, WA 6009, Australia; 12 Seqirus, Montreal, QC H9H 4M7, Canada

**Keywords:** adjuvanted, immunization, influenza, pediatric, parental acceptance, vaccine hesitancy

## Abstract

Adjuvanted trivalent influenza vaccine (aTIV) provides enhanced protection against seasonal influenza in children compared with nonadjuvanted trivalent influenza vaccine (TIV). This prospective cohort study assessed parental attitudes, beliefs, and intentions to vaccinate their infants aged 6–23 months with aTIV. Parents were surveyed before and after routine healthy baby visits, and post clinician interaction results were analyzed using multivariable logistic regression. Physicians at 15 community practice clinics and nurses at 3 public health clinics participated; 207 parents were surveyed. After clinician consultation, most parents considered immunization with aTIV to be safe (72.9%), effective (69.6%), and important (69.0%); most perceived support for vaccination from significant others (62.8%) and clinicians (81.6%); and 66.6% intended to vaccinate their infant with aTIV. Parental attitudes toward vaccinating their infant with aTIV were strongly correlated with perceptions of vaccine safety, efficacy, and importance, and these represented the strongest influence on intentions to vaccinate (odds ratio (OR) 79.25; 95% confidence interval (CI) 6.05–1037.50). Parental intentions were further influenced by perceived strength of clinician recommendation (OR 4.55, 95% CI 1.38–15.06) and social support for vaccination (OR 3.46, 95% CI 0.50–24.13). These findings may inform clinician approaches to parental education to ensure optimal seasonal pediatric influenza vaccination.

## 1. Introduction

An average of 12,200 hospitalizations and approximately 3500 deaths are attributable to influenza in Canada each year [1,2,3,4]. In the 2018–2019 season, the rate of hospitalizations among children 4 years and younger was 83 per 100,000 population, more than twice as high as all other age groups except adults 65 years and older (94 per 100,000). Importantly, a lack of prior exposure to influenza raises susceptibility to infection in young children who are immunologically immature [5]. This leads to excess emergency room visits and hospitalizations attributable to influenza in this age group [6]. In fact, severe influenza illness is often observed in children with no underlying comorbidities [7]. Children’s vulnerability to influenza and associated sequelae represents a pattern seen worldwide. Across 92 countries in a 2018 analysis, influenza-related mortality in children younger than 5 years ranged from 2.1 to 23.8 per 100,000 population, and the highest percentages of hospitalizations and death are among children younger than 2 years [8,9].

The Canadian National Advisory Committee on Immunization, the U.S. Advisory Committee on Immunization Practices, the World Health Organization, and the UK’s Joint Committee on Vaccination and Immunisation recommend influenza immunization and identify young children as a priority group for vaccination given their elevated risk of influenza-related complications [10,11,12,13]. In infants and young children, one dose of MF59^®^-adjuvanted trivalent influenza vaccine (aTIV; Fluad™, Seqirus UK Limited, Montreal, QC, Canada) has been shown to induce higher haemagglutination inhibition (HI) titers faster and with greater persistence than non-adjuvanted TIV, with consistently higher seroprotection rates at increased HI titer thresholds against both homologous and heterologous influenza strains [14]. The aTIV vaccine has also been shown to be well tolerated, with no pattern of associated serious AEs [15], and it was significantly more effective in preventing polymerase chain reaction (PCR)-confirmed influenza compared with TIV [16]. The superior immunogenicity and efficacy of the adjuvanted quadrivalent vaccine have also been demonstrated in a significantly mismatched influenza season [17].

Despite the well-documented health benefits of routine infant influenza immunization [18,19], there is mounting evidence of parental concerns around vaccines with respect to the safety, necessity, and benefits of recommended vaccines [20,21,22,23]. One study found that 13% of parents do not follow recommended vaccination schedules for their children, and a large proportion of parents currently following the recommended schedule have attitudes suggesting that they may be ambivalent and at risk of switching to suboptimal vaccine regimens [24]. A Canadian study found very low rates of clinician-administered influenza vaccination in children aged 6 through 23 months, despite a universal vaccination program and high primary care visit rates [25]. During the 2016–2017 influenza season, only 26.5% of Canadian children aged 6 months through 4 years were vaccinated for influenza [26]. Suboptimal rates of influenza immunization in children are observed in the US, the UK, and other settings worldwide [8,9].

In this study, a prospective cohort study assessed parental attitudes, beliefs, and intentions to vaccinate their infants aged 6–23 months with aTIV. Parents were surveyed before and after routine healthy baby visits, and post clinician interaction results were analyzed using multivariable logistic regression. The primary objective of this study was to assess parental attitudes, perceptions, and intentions to vaccinate their infants with aTIV after receiving information on the vaccine from a physician or other health care provider (HCP) during a routine healthy baby visit. Attitudes and perceived social support are the core constructs of the well-researched and well-validated Theory of Reasoned Action; they are typically strongly predictive of intentions to uptake health behaviors, and they are amenable to change [27,28,29]. Understanding parental perspectives concerning the safety, efficacy, and importance of pediatric influenza vaccination is essential to efforts to achieve seasonal influenza vaccination targets. Results from this study are intended to support clinician and parent education in efforts to strengthen pediatric influenza vaccination uptake across primary care and public health influenza immunization settings.

## 2. Materials and Methods

A prospective cohort survey design was employed during the 2015–2016 influenza season to explore attitudes, beliefs, and intentions concerning aTIV. While aTIV received approval from Canadian regulatory authorities in 2015, this study was conducted before the vaccine was available to clinical practice, and the investigators assessed parental intentions to vaccinate their infants with aTIV “when it becomes available.” The research protocol received ethics approval from the Western University Health Sciences Research Ethics Board, the Fraser Health Research Ethics Board, and IRB Services.

**Figure 1:**
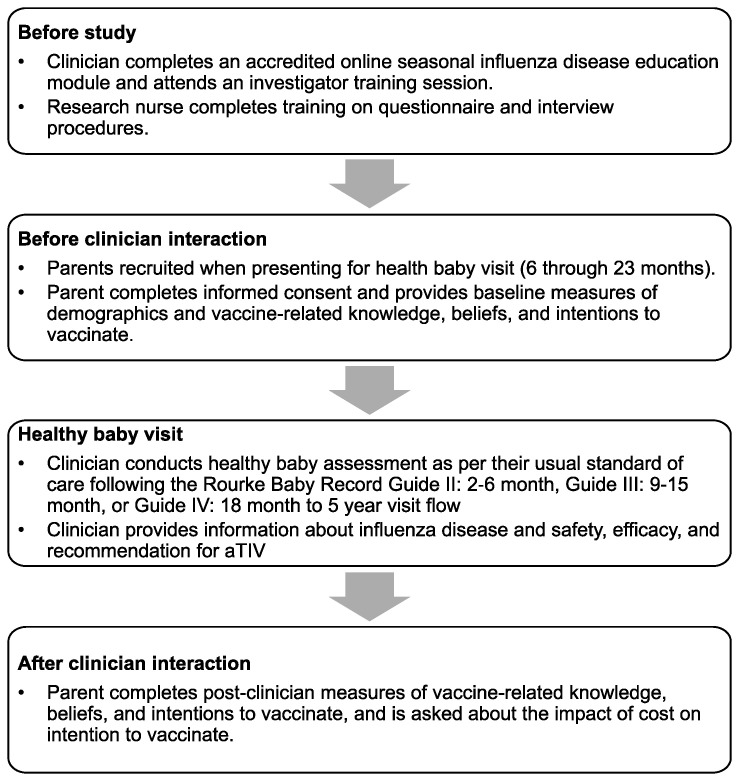
Study recruitment procedures and protocol flow.

The study population consisted of parents of infants aged 6 through 23 months, presenting for a scheduled healthy baby visit in the offices of participating pediatricians, family physicians, or nurses in primary care and public health settings in Canada. Participating parents were recruited and interviewed by a research nurse before and after a brief (<2 min) interaction with their clinician, in which information about influenza and aTIV was provided and the vaccine was recommended (Figure 1).

Eligible survey participants were the biological parent or legal caregiver of the infant, able and willing to provide written informed consent (in English or French), and able and willing to complete the two sets of questionnaires. Parents who had already participated in previous vaccine acceptance studies were excluded from this research to avoid the inclusion of pre-sensitized participants.

Participating parents were asked to complete two questionnaires that assessed their attitudes, perceptions, beliefs, and intentions with respect to infant immunization in general (in an initial pre-clinician interaction survey) and influenza disease and influenza vaccination in particular (in a post-clinician interaction survey). Data to address the current research focus concerning attitudes, beliefs, and intentions in relation to seasonal aTIV were taken from the post-clinician interaction assessment.

Descriptive statistics were first estimated for aggregated measures of the parameters of interest. Summary statistics including measures of central tendency, specifically the mean, median, standard deviation, and 95% confidence interval (CI) of the mean were estimated for continuous variables, and frequency distributions were estimated for categorical variables. Proportions expressing endorsement of vaccine-related intentions and beliefs (e.g., “I intend to vaccinate my baby with the adjuvanted seasonal flu vaccine when it becomes available”; “Getting my child the adjuvanted seasonal flu vaccination would be safe”) were coded on the basis of strong or moderate agreement on a 7-point Likert-type scale. Next, a multivariable logistic regression model was used to evaluate predictors of intention to vaccinate with the adjuvanted seasonal influenza vaccination. Individual predictors were chosen using a backwards selection method from univariable regression models. Variables were entered and removed at the alpha = 0.05 and alpha = 0.10 levels, respectively. Predictors considered in the model included parental attitudes, perceived social support, perceived clinician support, highest level of self-reported education, yearly household income, and previous infant vaccination experience.

## 3. Results

### 3.1. Sample Characteristics

A total of 18 community practice and 3 public health clinic sites across Canada participated; research nurses enrolled 136 parent participants from 15 independent community practice clinics, and 71 parent participants were enrolled from 3 public health clinics (N = 207). Table 1 details the sociodemographic background of parent participants.

### 3.2. Survey Results

After a brief HCP interaction in which information concerning pediatric influenza disease and the adjuvanted seasonal influenza vaccine was provided, most parents perceived vaccination of their infant with aTIV to be “safe” (72.9%), “effective” (69.6%), “important” (69.0%), “wise” (71.5%), and “good” (72.0%). Most parents perceived social support from significant others and clinician support for vaccination (62.8% and 81.6%, respectively), and 66.6% of parents intended to vaccinate their infant with aTIV when the vaccine “became available”. Parental attitudes toward vaccinating their infants with aTIV were calculated on the basis of parental perceptions that doing so would be safe, effective, important, good, and wise. Parental attitudes towards vaccinating their infants were the strongest influence on intentions to vaccinate with aTIV (odds ratio (OR) 79.25; 95% CI 6.05 to 1037.50) in the multivariable analysis. Parental intentions to vaccinate their infants with aTIV were further influenced by perceived strength of clinician recommendation (OR 4.55; 95% CI 1.38 to 15.06), perceived social support (OR 3.46; 95% CI 0.50 to 24.13), and higher income (OR 5.40, 95% CI 1.11 to 26.40) in the multivariable model (Table 2).

Additional items in the post clinician interaction survey provide further information relevant to seasonal pediatric influenza vaccine uptake. Approximately 1 out of 5 (20.3%) parents indicated that the necessity of returning to the clinic for any additional vaccinations would make it less likely that they would have their infant vaccinated against seasonal influenza. In addition, 17.4% indicated that having their child undergo an additional injection (to protect them against seasonal influenza) at their routine immunization visit would make it less likely that their child would receive their routine vaccinations. In contrast, 69.0% of parents indicated that they would permit their child to receive their routine vaccinations together with their seasonal influenza vaccination at the same visit, and 61.1% of parents indicated they would not be deterred from having their infant receive seasonal influenza vaccination if they had vaccine-site tenderness or a fever.

## 4. Discussion

The current research was conducted in the setting of suboptimal seasonal influenza vaccine coverage for young children who are subject to concerning levels of influenza-related sequelae. Our findings indicate that, after a brief interaction with a clinician who provides information about pediatric influenza and aTIV, most parents intended to vaccinate their infant with the vaccine when it became available. These findings are particularly noteworthy in the context of very low uptake rates for pediatric influenza vaccination in the Canadian setting, suboptimal uptake in many other locales, and heightened vaccine hesitancy around the world [8,9,20,21,22,23,26].

Attitudes towards vaccinating one’s infant with aTIV, based upon perceptions that this vaccine is safe, effective, and necessary, were the strongest single predictor of parental intentions to vaccinate their infant. Perceptions of social support from significant others (spouse, friends, family members, etc.), as well as perceptions of clinician support, also had significant and substantial relationships with parental intentions to vaccinate infants against influenza with an adjuvanted vaccine. Critically, each of these influential factors—perceived safety, efficacy, and importance of the vaccine; perceived social support for vaccination; and perceived HCP support for doing so—are amenable to change via targeted parent and clinician education. Such educational efforts would appear to be quite valuable in the setting of elevated pediatric risk of influenza complications coupled with low rates of seasonal pediatric influenza vaccination.

Additional information collected in this research is informative concerning the perceived impact of efforts to reinforce the inclusion of seasonal pediatric influenza vaccination into the routine vaccine schedule. When questioned, most parents believed that the inclusion of a seasonal pediatric influenza vaccination would not be problematic or interfere with other vaccination requirements, although a nontrivial proportion of parents—approximately 1 in 5—did express concerns and sensitivity to this issue. Parent and provider education, and scheduling flexibility when indicated, may help resolve these concerns.

The strengths of this study include the representation of healthy baby visits in primary care and public health settings, well-validated theoretical foundations, systematic assessment of intentions to vaccinate infants against influenza, and factors associated with strengthened intentions to do so. An additional strength of this research involves the brevity, feasibility, and impact of clinician–parent discussion of pediatric influenza and influenza vaccine protection. A primary limitation of this research involves the assessment of intentions to vaccinate as opposed to vaccination per se. Intentions have been identified as one of the strongest empirical predictors of future novel behaviors [27,28], and there is value in gathering information about vaccine acceptance prior to its widespread availability. Further research is needed to assess the specific predictive strength of these intentions, as well as factors that may positively influence vaccine intention and vaccine behavior concordance. We would add that in a companion publication (Fisher et al., in this issue), we have demonstrated that a similar, brief, feasible clinician–parent discussion resulted in distinctively elevated levels of actual influenza vaccination and choice of aTIV.

## 5. Conclusions

After a brief interaction with a clinician concerning pediatric influenza and influenza vaccination, most parents intended to vaccinate their infant with aTIV. This demonstration is particularly noteworthy in the context of elevated risk of influenza complications in young children, together with low influenza vaccination rates in the pediatric population. Vaccine uptake intentions were influenced by modifiable factors including perceptions of vaccine safety, efficacy, and importance; social support; and clinician support. Strengthening these perceptions through parent and clinician education would appear to be a worthy goal.

## Figures and Tables

**Table 1:** Sociodemographic characteristics.

Characteristic	Survey Population (N = 207)
Mean age (range)	
Parents (years)	33 (17–54)
Children (months)	13.5 (6–24)
Female sex, n (%)	
Parents	172 (83.1)
Children	101 (48.8)
Highest education level attained by parent, n (%)	
University (bachelor’s degree or higher)	106 (51.2)
Community college, technical college, or trade school	64 (30.9)
High school or equivalent	35 (16.9)
Primary school	2 (1.0)
Parental race and ethnicity, n (%)	
White	133 (64.3)
Asian	46 (22.2)
Native American	5 (2.4)
Black	6 (2.9)
Other	17 (8.2)

**Table 2:** Predictors of intention to vaccinate with the adjuvanted seasonal influenza vaccination (multivariate logistic regression model results).

Variable		Odds Ratio (95% CI)
Attitude	Negative	Referent
	Neutral	22.01 (6.12 to 79.19)
	Positive	79.25 (6.05 to 1037.50)
Social support	Negative	Referent
	Neutral	5.04 (1.48 to 17.13)
	Positive	3.46 (0.50 to 24.13)
Clinician support	Negative	Referent
	Neutral	1.36 (0.33 to 5.52)
	Positive	4.55 (1.38 to 15.06)
Income	≤$39,999	Referent
	$40,000–$79,999	4.49 (0.86 to 26.63)
	≥$80,000	5.40 (1.11 to 26.40)

Abbreviation: CI, confidence interval.

## Data Availability

The data presented in this study are available on request from the corresponding author.
